# Beta‐ketothiolase deficiency: A case with unusual presentation of nonketotic hypoglycemic episodes due to coexistent probable secondary carnitine deficiency

**DOI:** 10.1002/jmd2.12022

**Published:** 2019-03-14

**Authors:** Morteza Alijanpour, Hideo Sasai, Elsayed Abdelkreem, Yasuhiko Ago, Shima Soleimani, Leila Moslemi, Seiji Yamaguchi, Masomeh Rezapour, Mohammad T. Hakimi, Hideki Matsumoto, Toshiyuki Fukao

**Affiliations:** ^1^ Department of Pediatrics Non‐Communicable Pediatric Diseases Research Center, Health Research Institute, Babol University of Medical Sciences Babol IR Iran; ^2^ Department of Pediatrics, Graduate School of Medicine Gifu University Gifu Japan; ^3^ Department of Pediatrics, Faculty of Medicine Sohag University Sohag Egypt; ^4^ Babol Razi Pathobiology Laboratory Babol IR Iran; ^5^ Department of Pediatrics Shimane University School of Medicine Izumo Japan; ^6^ School of Traditional Medicine, Traditional Medicine and History of Medical Sciences Research Center, Health Research Institute, Babol University of Medical Sciences Babol IR Iran

**Keywords:** beta‐ketothiolase deficiency, case report, hypoglycemia, mitochondrial acetoacetyl‐CoA thiolase, secondary carnitine deficiency, T2

## Abstract

Beta‐ketothiolase (T2, mitochondrial acetoacetyl‐CoA thiolase) deficiency is an autosomal recessive disorder of isoleucine catabolism and ketone body metabolism that is characterized by increased urinary excretion of 2‐methylacetoacetate, 2‐methyl‐3‐hydroxybutyrate, and tiglylglycine. Most patients with T2 deficiency develop their first severe ketoacidotic events between 5 and 24 months of age. We encountered a case of T2 deficiency who developed the first hypoglycemic crisis without ketosis during her neonatal period and repeated such nonketotic hypoglycemic crisis during her infancy and early childhood. This is a very atypical clinical phenotype in T2 deficiency. We finally realized that she also has severe carnitine deficiency which might suppress beta‐oxidation resulting in nonketotic hypoglycemia. After carnitine supplementation, she actually developed episodes with ketonuria. Her carnitine deficiency was probably a secondary deficiency which is rare in T2 deficiency but if present, may modify the clinical manifestation of T2 deficiency from ketoacidotic events to hypoketotic hypoglycemic events.

## 1 INTRODUCTION

The defect of mitochondrial acetoacetyl‐CoA thiolase (T2), known as beta‐ketothiolase deficiency, is an inherited metabolic disorder of isoleucine catabolism and ketone body metabolism.^1^, ^2^, ^3^ T2 deficiency is clinically characterized with intermittent ketoacidotic episodes following intercurrent illnesses, such as gastroenteritis and upper respiratory infection. Clinical features of ketoacidotic crises include lethargy, hypotonia, dyspnea and/or polypnea, vomiting, and dehydration. In most cases, the age of onset of first clinical episodes is between 5 months and 2 years (median = 15 months).^4^ Blood glucose level varies during ketoacidotic crises in T2 deficiency, ranging from 0.6 to 23.3 mmol.^5^ However, hypoglycemia is, in general, rare in ketoacidotic events.^4^, ^5^

Herein, we experienced a T2‐deficient patient with multiple hypoglycemic episodes but no detectable urinary ketones. This is a very atypical phenotype of T2 deficiency. We finally concluded that this patient had severe carnitine deficiency, probably a secondary deficiency, which suppressed beta‐oxidation resulting in nonketotic hypoglycemia.

## 2 CASE PRESENTATION

A girl was born to healthy related Iranian parents at 34 weeks gestation with intrauterine growth restriction (IUGR) with a birth weight of 2 kg. She was immediately admitted to NICU due to poor feeding and grunting. Meconium aspiration syndrome was suspected. Her laboratory investigations revealed normal levels of blood gases and glucose (Table 1). The patient was then discharged with a good general appearance on 12th day of birth. However, on the 17th day of life, she had an episode of tachypnea with hypoglycemia (blood glucose 1.3 mmol/L) and elevated lactate level (4.9 mmol/L). Blood gas analysis and ammonia were normal (Table 1). She was treated with intravenous fluids containing glucose, and frequent feeding was advised. At the age of 53rd days, she was admitted again because of poor feeding, lethargy, and hypotonia with a suspicion of sepsis. Blood gas analysis, glucose, and blood counts were normal and urinary ketone was negative (Table 1). Urinary organic acid analysis performed by Austria Centogen lab showed increased excretion of 2‐methyl‐3‐hydroxybutyrate and tiglylglycine with normal level of 3‐hydroxybutyrate and acetoacetate. Dried blood spot (DBS) acylcarnitine analysis showed no elevation of C5OH and C5:1 carnitine levels. At that time, since urinary ketones were normal during her metabolic episode, she was tentatively suspected as having 2‐methyl‐3‐hydroxybutyric aciduria (MHBD) rather than T2 deficiency, although MHBD has an X‐linked trait.

**Table 1:** Patient's episodes and laboratory data

Age	Clinical manifestation	Blood gas			Blood glucose (mmol/L)	Urinary ketone	Serum acylcarnitines (nmol/ml)				Urinary organic acids			
		pH	pCO_2_ (mmHg)	HCO_3_ (mmol/L)			C0	C2	C5OH	C5:1	3HB	AcAc	2M3HB	TIG
0 day	poor feeding, grunting	7.38	36	21	4.6	(−)								
17 day	poor feeding, tachypnea	7.46	30	20	1.3	(−)								
53 day	poor feeding, lethargy	7.46	23	22	6.3	(−)								
21.5 months	polypnea	7.11	16	5	3.9	(−)	4.50	4.50	0.25	0.18	ND	ND	147.9	202.7
24 months	polypnea	7.23	29	12.4	1.9	(−)								
2 years and 4 months	lethargy	7.36	31.7	18	1.6	(−)								
3 years and 5 months	anorexia, apathy	7.32	31	16.3	1.9		3.84	4.06	0.59	0.29	ND	ND	111.7	202.7
l‐carnitine supplementation 50 mg/kg/day														
3 years and 10 months	fever, apathy	7.36	27.9	15.8	3.1	(−)								
4 years and 1 month	polypnea	7.2	20	8	3.7	3+					2003.0	919.0	268.0	73.0
4 years and 4 months	gastroenteritis	7.32	16.3	8.4	2.4	1+	4.49	5.81	0.14	0.08	1677.4	137.4	88.6	45.2
l‐carnitine supplementation 100 mg/kg/day														
5 years and 2 months	stable						10.11	4.25	0.35	0.06	6.95	0.1	79.9	85.3
					normal range		7.44‐77.6	4.3‐56.6	<0.9	0.54‐1.53	<5.28	<0.1	<7.65	<0.53

Urinary organic acid analysis: the values are relative amounts to internal standard.

2M3HB, 2‐methyl‐3‐hydroxybutyrate; 3HB, 3‐hyroxybutyrate; AcAc, acetoacetate; TIG, tiglylglycine.

The girl again developed respiratory distress and lethargy at the age of 21.5 months. She had failure to thrive and her weight was only 7 kg. Laboratory findings showed severe metabolic acidosis (pH: 7.11, pCO_2_: 16.1 mmHg, HCO_3_: 5.1 mmol/L) and normal blood glucose (3.9 mmol/L). Urinary ketone was also negative (Table 1). Metabolic analyses were repeated due to severe metabolic acidosis (from this point, metabolic analyses were done by Razi lab of Iran). Results of urinary organic acid and blood analysis were analogous to the previous ones. At 24, 28, and 41 months of age, she developed repeated similar episodes of respiratory distress and lethargy; her laboratory tests showed mild metabolic acidosis and hypoglycemia with negative urinary ketone (Table 1).

The Department of Pediatrics at Gifu University was consulted about this patient to perform genetic analysis of MHBD and T2 deficiency. The diagnosis of T2 deficiency was confirmed by *ACAT1* mutation analysis. However, we could not explain why she had no ketosis even during serious conditions. Since the parents were related, our first hypothesis was the coexistence of another genetic disorder which causes hypoketotic hypoglycemia. Accordingly, we performed DNA panel analysis for defects in beta‐oxidation, carnitine cycle disorders and glycogenolysis. However, no significant variants were identified.

At that moment, we re‐evaluated previous metabolic analyses and realized that her free carnitine level was very low. The low free carnitine level was confirmed by blood acylcarnitine analysis at age of 3 years and 5 months. In addition to frequent feeding, l‐carnitine supplementation (50 mg/kg/day) was started. As shown in Table 1, after carnitine supplementation, she had three mild episodes, two of which were associated with ketonuria. At the age of 4 years and 5 months, l‐carnitine dose was increased to 100 mg/kg/day. Results of repeated echocardiography and electrocardiography showed no remarkable abnormalities, and blood levels of creatine phosphokinase and liver transaminases were within normal range.

Now, she is 5‐years and 5‐month‐old and her height is 94 cm (−3.68 SD), and her weight is 12 kg (−3.51 SD), indicating persistent failure to thrive. Endocrine evaluation was normal. Her neurological development is normal.

Reviewing the nutritional history showed that the patient had breast‐fed for the first 2 months of life, then she had artificial formula. In the first 3 years of life, she was highly restricted in taking animal proteins (such as cow milk and dairy products). Even now she eats a small amount of meats or fish once a week; she prefers fruits and vegetables.

She has one sister with normal IQ. Her weight and height are below the 3rd percentile in growth curve. Father's and mother's heights were 160 cm (−2.27 SD) and 150 cm (−2.01 SD), respectively.

## 3 MATERIALS AND METHODS

### 3.1 Mutation analysis

Genomic PCR followed by direct sequencing for *ACAT1* and *HSD17B10* were done as described.^6^, ^7^ We also performed mutation analysis using a DNA panel consisting of 59 genes which may be involved in fatty acid oxidation, ketone body metabolism and transport, and glycogen storage diseases using the MiSeq Sequencing System (Illumina, San Diego, CA) at the Kazusa DNA Research Institute. This panel included *SLC22A5* for primary carnitine deficiency. Transient expression analysis of wild‐type and mutant cDNAs were done as described.^8^

## 4 RESULTS

We identified a homozygous mutation of c.1035‐1037delAGA (p.Glu345del) in *ACAT1* gene, which was previously reported.^9^ Expression analysis of mutant cDNA showed no residual T2 activity. No *HSD17B10* mutation was identified.

DNA panel analysis detected no causative mutations in the genes for defects in beta‐oxidation (*ACADVL*, *ACADM*, *HADHA*, *HADHB*) and carnitine cycles (*CPT1A*, *SLC25A20*, *CPT2*), and carnitine uptake (*SLC22A5*).

## 5 DISCUSSION

T2 deficiency is an inborn error of ketone body utilization and isoleucine catabolism. This disorder is clinically characterized by intermittent ketoacidotic crises and no symptom during nonepisodic condition. Ketogenic stresses, such as fever, vomiting and fasting, usually precipitate such metabolic crises. During metabolic episodes, almost all T2‐deficient patients have ketoacidosis.^4^ However, clinical manifestation of our T2‐deficient patient was highly unusual. She developed repeated hypoglycemic episodes with negative urinary ketone. This hypoketotic hypoglycemia is a characteristic feature for defects in ketogenesis and beta‐oxidation but not for defects in ketolysis such as T2 deficiency.^1^, ^2^, ^3^, ^4^, ^5^

Molecular study clearly showed that our patient has T2 deficiency. She is a homozygote of p.Glu345del, which was already identified in two other patients and we confirmed by mutant cDNA expression analysis that this mutation had no residual T2 activity. However, T2 deficiency alone cannot explain her hypoketotic hypoglycemic episodes. We did DNA panel analysis for primary carnitine deficiency, beta‐oxidation defects, defects in carnitine cycle, and glycogenolysis. No possible mutations were identified.

As shown in Table 1, we finally realized that free carnitine levels were very low in this patient. Such levels of low free carnitine are similar to those in patients with primary carnitine deficiency^10^ or patients with secondary carnitine deficiency caused by antibiotics containing pivalic acid, who developed hypoketotic hypoglycemia.^11^, ^12^ Hence, carnitine deficiency may explain why this patient had multiple hypoglycemic episodes with negative urinary ketone despite having T2 deficiency. In case of genetic analysis of *SLC22A5* for primary carnitine deficiency, in addition to routine mutation analysis, copy number variation analysis was also done. However, we could not find any possible mutations. This indicates that she likely has a secondary carnitine deficiency, although we could not absolutely rule out primary carnitine deficiency, including its heterozygous condition by such molecular analyses.^13^ Unfortunately, her fibroblasts are not available for functional analysis. Accordingly, the patient received carnitine supplementation (50 mg/kg/day), after which her urinary ketone became positive during subsequent milder hypoglycemic episodes with metabolic acidosis. Eleven months after starting carnitine therapy, her blood free carnitine level was still low. Hence, carnitine supplementation was increased to 100 mg/kg/day.Severe carnitine deficiency hinders carnitine‐conjugation of accumulated short chain acyl‐CoAs from isoleucine catabolism and impairs intramitochondrial metabolism by free CoA restriction. Carnitine deficiency also affects beta‐oxidation by insufficient carnitine cycle function.^9^ We speculate that this explains why this patient developed multiple metabolic episodes.

In this patient, as discussed above, we could not rule out completely the possibility that she is affected with primary carnitine deficiency since we could not perform functional assays.^12^ However, there are several factors which might cause secondary carnitine deficiency. She was born with IUGR (birth weight 2 kg). She likes fruits and vegetables for diet and only eats small amount of meat and fish. Since carnitine is contained in meats and fish, her food preference may be associated with her carnitine deficiency. Carnitine could be consumed by urinary excretion of 2‐methyl‐3‐hydroxycarnitine and tiglylcarnitine by T2 deficiency like other urinary organic acidemias. Finally, she had continuous failure to thrive. This is also unusual for T2 deficiency, and it is difficult to explain such severe failure to thrive by T2 deficiency or secondary carnitine deficiency. Given that her family members also exhibit short stature, another genetic factor may contribute to her short stature. In conclusion, we presented a case of T2‐deficient patient with atypical manifestation of multiple hypoketotic hypoglycemic episodes. Although T2 deficiency is usually characterized by intermittent ketoacidotic crises, hypoketotic hypoglycemia was prominent as a clinical manifestation of coexistent carnitine deficiency, of which cause is unknown.

## CONFLICTS OF INTEREST

The authors have no conflicts of interest to report.

## AUTHOR CONTRIBUTIONS

Morteza Alijanpour Aghamaleki, Leila Moslemi, and Masomeh Rezapour were involved in clinical management of patients and wrote the first draft. Shima Soleimani Amiri, Masomeh Rezapour, and Mohammad Taghi Hakimi were involved biochemical analyses of this patient. Elsayed Abdelkreem, Hiroki Otsuka, Hideo Sasai, and Hideki Matsumoto performed mutational and expression analyses. Toshiyuki Fukao initiated and supervised this study, reviewed and revised the manuscript, and approved the final version as submitted. All authors approved the final manuscript as submitted and agree to be accountable for all aspects of the work. All authors confirm the absence of previous similar or simultaneous publications.

## ETHICS APPROVAL

This study was approved by the Ethical Committee of the Graduate School of Medicine, Gifu University, Japan, and was carried out in accordance with the principles contained within the Declaration of Helsinki. Informed consents were obtained from the parents.
